# Comparative analysis of transvaginal and transabdominal sliding sign for predicting intra-abdominal adhesions prior to repeat cesarean section: a single center study

**DOI:** 10.7717/peerj.20551

**Published:** 2026-01-09

**Authors:** Peby Maulina Lestari, Imas Kartika Dewi E, Abarham Martadiansyah, Theodorus Theodorus, Nuswil Bernolian, Putri Mirani, Muhammad Al Farisi Sutrisno, Bella Stevanny

**Affiliations:** 1 Department of Obstetrics and Gynecology, Dr. Mohammad Hoesin General Hospital, Faculty of Medicine, Sriwijaya University, Palembang, South Sumatra, Indonesia; 2 Department of Pharmacology, Sriwijaya University, Palembang, South Sumatra, Indonesia

**Keywords:** Caesarean section, Intraabdominal adhesion, Sliding sign, Transabdominal ultrasound, Transvaginal ultrasound

## Abstract

**Background:**

Caesarean section plays a crucial role in ensuring the health of both mother and newborn, especially when complications arise or are anticipated. However, the increasing global prevalence of caesarean section brings along significant postoperative challenges, notably pelvic adhesions, which can impact subsequent pregnancies and surgeries. Non-invasive preoperative assessment methods, such as ultrasonography, offer promise in enhancing surgical planning and patient outcomes.

**Objective:**

This study aims to evaluate the accuracy of transabdominal and transvaginal sliding sign in detecting intraabdominal adhesions in third-trimester pregnant women with a history of caesarean section.

**Method:**

This diagnostic study recruited 35 third-trimester pregnant women with a history of cesarean section undergoing scheduled repeat cesarean at a tertiary referral hospital. All participants underwent both via transvaginal (TV) and transabdominal (TA) ultrasound before surgery. The presence of a positive or negative sliding sign was recorded for each modality. Intra-abdominal adhesions were confirmed intraoperatively and used as the gold standard. Diagnostic accuracy was calculated for each modality. Comparative analysis was conducted across patient characteristics and outcomes.

**Result:**

All 35 patients underwent both TA and TV ultrasound evaluations (within-subject design). The majority of participants were aged between 31 and 40 years (68.6%), were multigravida (65.7%), and had undergone two caesarean sections (51.4%). The sliding sign was negative in 18 (51.4%) and 17 (48.6%) patients using TA and TV approaches, respectively. Both modalities showed high sensitivity (93.75%) and specificity (84.21%) in detecting adhesions. There was no statistically significant difference between TA and TV sliding signs in relation to age or obstetric status. Comparative analysis of both modalities showed equivalent diagnostic performance.

**Conclusion:**

Both transabdominal and transvaginal sliding sign evaluations demonstrate high accuracy in detecting intra-abdominal adhesions in women undergoing repeat cesarean section. The within-subject comparison suggests either modality may be reliably used depending on clinical and logistic factors.

## Introduction

Cesarean section (CS) is the most commonly performed major surgical procedure worldwide and continues to increase in frequency across both low- and high-resource settings. According to the World Health Organization, cesarean delivery rates have reached over 21% globally, with some countries reporting rates exceeding 40%. This trend has raised important concerns regarding the long-term maternal consequences of repeated CS, including the formation of intra-abdominal adhesions (Boerma et al., 2018; Biccard et al., 2018). Intra-abdominal adhesions are fibrous bands that form between tissues and organs, often as a result of surgical trauma. Predominantly, these adhesions manifest within the vesicouterine pouch, situated between the uterus and the anterior abdominal wall (Moro et al., 2015). Intra-abdominal adhesions occur in up to 74% of women undergoing repeat cesarean delivery and are associated with increased operative time, accidental injury to bowel or bladder, excessive bleeding, and challenges in future pelvic surgeries or fertility procedure (Pokhrel et al., 2022). Despite their clinical importance, adhesions remain difficult to detect non-invasively prior to surgery.

The “sliding sign” is a dynamic ultrasonographic feature that assesses the gliding movement between the uterus and the anterior abdominal wall during maternal respiration. It has emerged as a promising, simple, non-invasive tool for preoperative prediction of adhesions. The noninvasive detection of pelvic adhesions following CS in an outpatient setting presents several significant benefits. For symptomatic women not intending pregnancy, it allows for adhesion identification and subsequent counseling on management strategies. Additionally, informing the obstetrician about extensive adhesions preoperatively enables better surgical planning and enhances operative safety (Holland et al., 2010; Hudelist et al., 2009). The sliding sign ultrasonography technique in assessing pre-operative adhesions can be performed both transabdominally and transvaginally. A previous study by Ayachi et al. (2018) reported transvaginal sliding sign had a sensitivity of 96.3%, specificity 92.6% to predict adhesions. However, the transvaginal route may not always be acceptable or feasible in all obstetric settings, particularly during the third trimester. Transabdominal ultrasound, which is more routinely performed during pregnancy, could provide a more accessible alternative. Nevertheless, data comparing the accuracy of transabdominal and transvaginal sliding signs in predicting adhesions prior to repeat cesarean delivery remain limited, especially in Southeast Asian populations. The ability to predict severe adhesions based on non-invasive sliding sign examination will provide safer surgical planning and techniques for patients with a history of surgery (Stocker, Glazebrook & Cheong, 2014). This study aimed to evaluate and compare the diagnostic accuracy of transabdominal and transvaginal sliding signs for predicting intra-abdominal adhesions in third-trimester pregnant women with a history of cesarean section. Our outcome measures included sensitivity, specificity, positive predictive value (PPV), negative predictive value (NPV), and overall accuracy for both ultrasound modalities. Furthermore, we explored potential clinical markers, including incision type and keloid formation, which may correlate with the presence of adhesions. This research seeks to bridge a gap in current knowledge by identifying reliable preoperative tools that may reduce surgical complications and optimize obstetric care.

## Materials & Methods

This is a diagnostic accuracy study with a within-subject design on third trimester pregnant women who have a previous history of caesarean section and are planned to undergo caesarean delivery in the Obstetrics and Gynecology Department of Mohammad Hoesin Hospital Palembang. Patients with placenta previa/accreta, multiple pregnancy, uterine anomaly, and emergency CS were excluded from the study. The recruitment period of this study started from September 1st 2020 to August 31st 2021. The minimum sample size was calculated using Lemeshow formula using confidence level of 95%, power level of 90%, and intraabdominal adhesion following CS prevalence rate of 84.21% from Hudelist et al. (2009) was 35 subjects. Written informed consent for participation in the study was obtained from all subjects. All participants underwent both *via* transvaginal (TV) and transabdominal (TA) ultrasound performed consecutively by experienced maternal-fetal medicine specialists (≥10 years of experience in obstetric ultrasonography), who was blinded to the intraoperative findings. This uniformity ensured consistency and minimized inter-operator variability. A GE Voluson™ P8 ultrasound machine (GE Healthcare, Oberösterreich, Austria) equipped with both a convex abdominal probe (2–5 MHz) and an endovaginal probe (5–9 MHz) was used to perform the transabdominal and transvaginal sliding sign assessments, respectively. Each patient served as her own control, allowing direct comparison of the two modalities within the same clinical conditions. Data matching was carried out based on the variables age, obstetric status, and history of CS.

**Figure 1:**
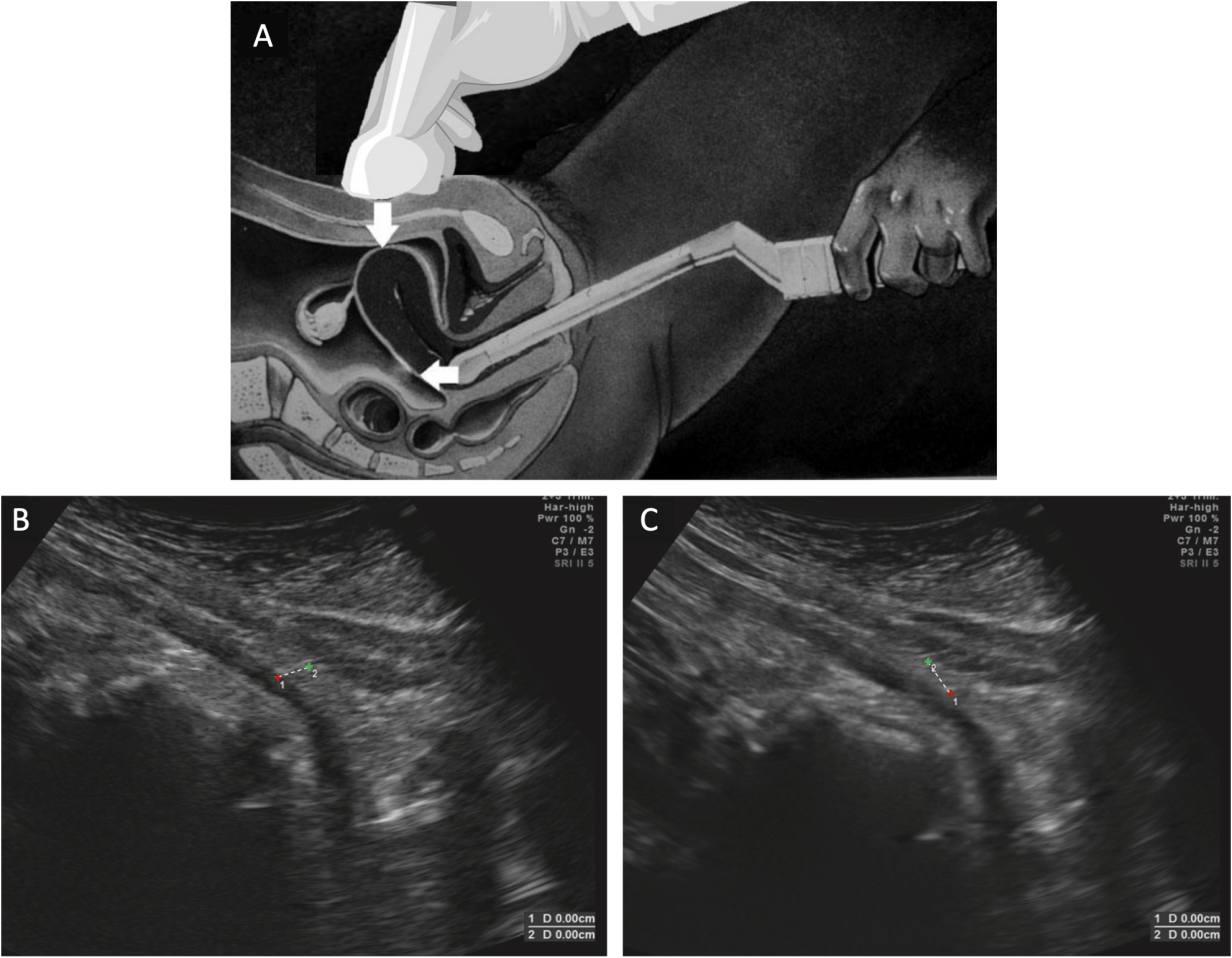
Sliding sign assessment using dynamic transvaginal sonography. (A) Transvaginal probe is inserted into the vaginal and then placed in the front wall of the vagina using gentle pressure and release motions in order to observe movement between the vesicouterine fold and the anterior uterine wall. Transabdominal probe is placed directly above perpendicular to the caesarean section incision mark. During examination, the probe was slightly moved back and forth along the scar and perpendicular to it. (B) The initial position before the patient was instructed to breathe; point 1 is located on the anterior uterine wall and point 2 is on the muscle fascia tissue. (C) The presence of uterine displacement against the abdominal wall was confirmed by a positive sliding sign. The position of point 1 on the anterior uterine wall has shifted downwards whereas point 2 on the muscular fascia has remained in its original position during the patient’s breathing. Modified with permission from Reid & Condous (2013) and Baron et al. (2018).

The sliding sign was evaluated according to standardized criteria. For transabdominal ultrasound examination, the probe was placed directly above perpendicular to the CS incision mark. During examination, the probe was slightly moved back and forth along the scar and perpendicular to it. The patient was then asked to take a deep breath to see the sliding or shift of the uterus caudally under the parietal peritoneum and transversalis fascia. Uterine displacement against the abdominal wall indicated a positive sliding sign. When there is no movement of the structures mentioned above, the sliding sign is considered negative. Transvaginal ultrasound examination was done by placing the transvaginal probe on the anterior vaginal wall with slow pressing and releasing movements to see movement between the vesicouterine fold and the anterior uterine side. A structure experiencing “sliding” would swing easily and indicated signs of positive sliding. The uterine fundus could also be mobilized with the hands to maximize swing. The probe was then placed again in front of the cervix, applying pressure to the fundus and mobilizing the fundus towards the intestine. If the intestine appears to swing towards the posterior fundus, then the sliding sign was considered positive (Fig. 1) (Baron et al., 2018; Reid & Condous, 2013). The presence of adhesion was confirmed intraoperatively. Intraoperative findings were recorded as the reference standard for the presence or absence of adhesions.

Comparative analyses were conducted using Chi-square and Fisher’s exact test. Diagnostic values were calculated using MedCalc Version 19 to determine the diagnostic value of sliding signs in the diagnosis of adhesions. In addition to the primary diagnostic accuracy outcomes, we also recorded incision type and the presence of keloid formation as secondary exploratory variables. These analyses were not part of the primary study objectives but were included *post hoc* to assess potential associations with intra-abdominal adhesions. Ethical permission was obtained for the study from the Ethics Committee of the Faculty of Medicine, University of Sriwijaya (616/kepkrsmhfkunsri/2019).

## Results

A total of 35 pregnant women in the third trimester with a history of CS who were planned to undergo CS delivery were included in the study. The general characteristics of the subjects were presented in Table 1. The mean age of the research subjects was 32.9 ± 4.89 years and the majority had an age range of 31–40 years (68.6%). Based on obstetric status, the majority of subjects were multigravida (68.6%) and 34.3% of subjects were grandemultigravida. The majority of the patients had two previous history of CS (53.4%). The proportion of negative sliding signs was 51.4% (transabdominal) and 48.6% (transvaginal). There were no significant differences in age or obstetric status across sliding sign results in both groups. However, the number of previous cesarean sections was significantly associated with adhesion status (*p* = 0.030), with adhesions more frequently observed in patients with two prior cesarean deliveries.

**Table 1:** Subject characteristics.

**Characteristic**	*n*(%)	**Adhesions**			**Keloid**		
		**Negative** (*n* = 16)	**Positive** (*n* = 19)	*P* value	**Negative** (*n* = 15)	Positive (*n* = 20)	*P* value
Age, mean ± SD (years)	32.9 ± 4.89			0.258^*^			0.633^*^
31–30 years old	9 (25.7%)	2 (12.5)	7 (36.8)		5 (33.3)	4 (20.0)	
31–40 years old	24 (68.6%)	13 (81.3)	11 (57.9)		9 (60.0)	15 (75.0)	
>40 years old	2 (5.7%)	1 (6.3)	1 (5.3)		1 (6.7)	1 (5.0)	
Obstetric status				0.090^**^			0.034^**^
Multigravida (2–4)	23 (65.7%)	8 (50.0)	15 (78.9)		13 (86.7)	10 (50.0)	
Grandemultigravida (≥5)	12 (34.3%)	8 (50.0)	4 (21.1)		2 (13.3)	10 (50.0)	
Previous history of CS				0.030^*^			0.020^*^
1	5 (14.3%)	0 (0)	5 (26.3)		4 (26.7)	1 (5.0)	
2	18 (51.4%)	7 (43.8)	11 (57.9)		10 (66.7)	8 (40.0)	
3	11 (31.4%)	8 (50.0)	3 (15.8)		1 (6.7)	10 (50.0)	
4	1 (2.9%)	1 (6.3)	0 (0)		0 (0)	1 (5.0)	

**Notes.**

* Pearson Chi square. *p* = 0.05.

** Fisher exact test. *p* = 0.05.

Table 2 shows the comparison between transabdominal and transvaginal sliding sign results. The transabdominal ultrasound identified 16 cases with negative sliding signs and one case with a positive sliding sign among the 17 participants confirmed to have adhesions. Conversely, transvaginal ultrasound depicted a similar pattern with 16 negative and one positive sliding signs in the same group. For participants without adhesions, both techniques showed one negative and 17 positive sliding signs, respectively. There is excellent agreement between transabdominal and transvaginal sliding sign findings. Out of 35 cases, 33 (94.29%) showed concordant results between both modalities. The Cohen’s kappa coefficient was 0.89 indicating almost perfect agreement between the two ultrasound techniques, suggesting that both modalities are highly consistent with each other in detecting intra-abdominal adhesions.

**Table 2:** Comparison between transabdominal and transvaginal sliding sign results.

Examination		**Transvaginal** ** sliding sign**		**Total**	**Overall agreement**	**Cohen’s Kappa (standard error)**
		**Negative**	**Positive**			
**Transabdominal sliding sign**	**Negative**	16	1	17		
	**Positive**	1	17	18	94.29%	0.886 (0.079)
**Total**		17	18	35		

For both transvaginal and transabdominal sliding signs, a positive sliding sign was significantly associated with absence of adhesions, while a negative sliding sign was strongly associated with presence of adhesions (Table 3). Out of 16 patients with adhesions, 15 (93.75%) had a negative sliding sign, and only one (6.25%) had a positive sliding sign. Conversely, among the 19 patients without adhesions, 16 (84.21%) had a positive sliding sign, and three (15.79%) had a false negative result (negative sliding sign without adhesions). The statistical analysis yielded a highly significant association (*p* < 0.001) for both modalities. The odds ratio (OR) was 80.00, with a 95% confidence interval (CI) of 7.47 to 856.01, indicating that a patient with a negative sliding sign is 80 times more likely to have adhesions compared to a patient with a positive sliding sign. Despite the wide confidence interval—likely due to the modest sample size—the statistical strength (*p* < 0.001) remains robust.

**Table 3:** Association between pre-operative sliding sign findings and intraabdominal adhesions on intraoperative evaluation.

**Variables**	**Adhesion**		***p*-value**	**OR**
	Yes (*n* = 16)	No (*n* = 19)		
Transvaginal sliding sign				80.00 (7.47–856.01)
Negative	15	3	<0,001^a^	
Positive	1	16		
Transabdominal sliding sign				80.00 (7.47–856.01)
Negative	15	3	<0.001^a^	
Positive	1	16		

**Notes.**

OR, odds ratio.

a Pearson Chi-square test.

b Fisher’s exact test.

Adhesions were intraoperatively confirmed in 16/35 cases (45.7%). The diagnostic performance of transabdominal and transvaginal sliding sign techniques for detecting intra-abdominal adhesions was evaluated and compared (Table 4). Both transabdominal and transvaginal sliding signs demonstrated identical and excellent diagnostic performance in predicting intra-abdominal adhesions. The area under the curve (AUC) was 0.890 (95% CI [0.770–1.000]) for both modalities, indicating high overall accuracy. The sensitivity was 84.21% (95% CI [62.43–94.48]), and the specificity reached 93.75% (95% CI [71.67–98.89]), reflecting the sliding sign’s robust ability to detect true positive and true negative cases. The positive predictive value (PPV) was 83.33% (95% CI [60.78–94.16]), while the negative predictive value (NPV) was 94.12% (95% CI [73.02–98.95]), suggesting strong reliability in excluding adhesions when the sign is positive. Furthermore, the positive likelihood ratio (PLR) was 5.96 (95% CI [2.09–16.90]), and the negative likelihood ratio (NLR) was 0.07 (95% CI [0.03–0.21]), indicating excellent diagnostic utility. The overall diagnostic accuracy of both approaches was 88.57% (95% CI [74.05–95.46]). These findings highlight that both TA and TV sliding sign are robust predictors of intra-abdominal adhesions, and their diagnostic accuracy appears equivalent in this study population. This equivalence suggests that the selection between these two diagnostic modalities can be tailored based on patient-specific factors, procedural preferences, or resource availability, without compromising diagnostic integrity.

**Table 4:** Comparative diagnostic performances of transabdominal and transvaginal sliding signs in predicting intra-abdominal adhesions.

**Diagnostic value (95% CI)**	**Transabdominal sliding sign**	**Transvaginal sliding sign**
AUC	0.890 (0.770–1.000))	0.890 (0.770–1.000))
Sensitivitas (%)	84.21 (62.43–94.48)	84.21 (62.43–94.48)
Spesifisitas (%)	93.75 (71.67–98.89)	93.75 (71.67–98.89)
PPV (%)	83.33 (60.78–94.16)	83.33 (60.78–94.16)
NPV (%)	94.12 (73.02–98.95)	94.12 (73.02–98.95)
PLR	5.96 (2.09–16.90)	5.96 (2.09–16.90)
NLR	0.07 (0.03–0.21)	0.07 (0.03–0.21)
Accuracy (%)	88.57 (74.05–95.46)	88.57 (74.05–95.46)

**Notes.**

95% CI 95% confidence interval AUC area under the curve PPV positive predictive value NPV negative predictive value PLR positive likelihood ratio NLR negative likelihood ratio

In the analysis of incision type, 13 out of 16 patients (81.3%) with adhesions had previously undergone Pfannenstiel incisions, while only three patients (18.8%) had adhesions after mediana incisions. Despite this distribution, the association between incision type and intra-abdominal adhesions was not statistically significant (*p* = 0.312) with an odds ratio (OR) of 4.154 (95% CI [0.387–44.566]), indicating wide variability and a potential lack of power to detect significance due to small subgroup sizes (Table 5).

**Table 5:** Association between incision type and intraabdominal adhesions.

**Examination**		**Adhesion**		**Total**	***P* value**	**OR**
		**Positive**	**Negative**			
**Incision type**	**Mediana**	3	1	4	
	**Pfannenstiel**	13	18	31	0.312	4.154 (0.387–44.566)
**Total**		16	29	35		

**Notes.**

Pearson chi square, *p* = 0.05.

Conversely, keloid formation showed a strong and statistically significant association with intra-abdominal adhesions. Among patients with keloids, 14 of 20 (70%) had adhesions, compared to only two of 15 (13.3%) in the non-keloid group. This difference was highly significant (*p* < 0.001) with an odds ratio of 15.167 (95% CI [2.585–86.990]), suggesting that patients with keloid-prone skin are over 15 times more likely to also have intra-abdominal adhesions (Table 6). In this study, there were 15 subjects with negative keloids and 20 patient subjects with positive keloids. With statistical analysis, it was found that there was no difference in age (*p* = 0.633) between patients with positive and negative keloids. However, there were differences in obstetric status (*p* = 0.034) and history of CS (*p* = 0.020) between patients with positive and negative keloids (Table 1).

**Table 6:** Association between keloid formation and intraabdominal adhesions.

**Examination**		**Adhesion**		**Total**	***P* value**	**OR**
		**Positive**	**Negative**			
**Keloid**	**Positive**	14	6	20		
	**Negative**	2	13	15	<0.001	15.167 (2.585–86.990)
**Total**		16	19	35		

**Notes.**

Pearson Chi square *p* = 0.05.

## Discussion

This study aimed to assess the diagnostic accuracy of the sliding sign *via* transvaginal and transabdominal ultrasound in predicting intra-abdominal adhesions in third-trimester pregnant women with a history of CS. Adhesions were defined intraoperatively as fibrous bands of scar tissue that abnormally tethered the uterus to adjacent organs or the anterior abdominal wall, limiting tissue mobility and altering expected anatomic planes. This definition was based on prior studies by Drukker et al. (2018) and Ayachi et al. (2018) in which intraoperative visual confirmation of dense connective tissue replacing the normal peritoneal sliding plane was considered diagnostic. From a surgical perspective, intraoperative identification of adhesions was based on gross visualization of fibrous bands, tissue bridging, or immobility between the uterus and anterior abdominal wall, bladder, or bowel—consistent with definitions used in previous study by Drukker et al. (2018). Although we did not use a formal adhesion scoring system, *e.g.*, the peritoneal adhesion index or Zühlke classification, the binary classification of adhesion presence remains a clinically relevant endpoint, particularly when considering operative difficulty, blood loss, bladder dissection time, and uterine exteriorization.

Both transabdominal and transvaginal ultrasound demonstrated high diagnostic performance in predicting adhesions, with identical values for sensitivity (93.75%), specificity (84.21%), PPV (83.3%), NPV (94.1%), and overall accuracy (88.6%). These findings are comparable to previous studies. Previous studies have supported the sliding sign as an effective predictor of pelvic or intra-abdominal adhesions. Mayibenye et al. (2024) performed a large prospective observational study in Nigeria involving 419 women with a history of cesarean section. They evaluated both paraumbilical and suprapubic transabdominal sliding signs, reporting excellent diagnostic performance in predicting dense intra-abdominal adhesions, with sensitivities of 94.6% and 95.2% and specificities of 93.3% and 91.7%, respectively. Their results affirm the applicability of the sliding sign in high-parity, low-resource settings and align closely with our findings. Similarly, Taha et al. (2025) confirms the predictive validity of the transabdominal sliding sign for intra-abdominal adhesions before repeat cesarean section. Their work underscores the growing consensus that dynamic ultrasonography offers a practical, accurate, and cost-effective tool for preoperative adhesion risk stratification, especially in women with a prior history of surgical intervention. Baron et al. (2018) introduced the third-trimester transabdominal sliding sign and reported a specificity of 95% for predicting adhesions prior to cesarean section, although the sensitivity was lower at 56%. Similarly, Ayachi et al. (2018) demonstrated that the transvaginal sliding sign has a sensitivity of 96.3% and specificity of 92.6% in detecting adhesions in patients with prior abdominopelvic surgery. Menakaya et al. (2013) highlighted the sliding sign’s utility when combined with sonovaginography, particularly in patients with deep infiltrating endometriosis. A recent systematic review and meta-analysis by Shafti et al. (2023) pooling 25 studies (≈ 1,840 patients with adhesions, 2,501 controls) evaluated predictors of intraperitoneal adhesions after repeat cesarean sections. It found that a negative sliding sign yielded sensitivity of 0.71 (95% CI [0.65–0.77]) and specificity of 0.87 (95% CI [0.85–0.89]) (Shafti et al., 2023). Our study further supports these findings, showing that both TA and TV ultrasound modalities perform similarly well in third-trimester obstetric populations. These findings collectively support the value of the sliding sign as a non-invasive, reproducible bedside tool to anticipate operative challenges and enhance surgical preparedness in repeat cesarean cases.

The sliding sign technique is based on the physiological principle that normal intra-abdominal organs glide freely across each other due to a smooth, frictionless peritoneal surface. When adhesions are present, this movement is restricted. In TA ultrasound, the uterus is expected to shift caudally during deep inspiration, separating from the anterior abdominal wall; while in TV ultrasound, gentle pressure applied *via* the vaginal probe allows assessment of the mobility of the uterus against the bladder or adjacent bowel. The absence of this mobility—*i.e.*, a negative sliding sign—is indicative of peritoneal scarring or fibrous bridging that tethers the uterus to adjacent structures (Ayachi et al., 2018; Baron et al., 2018). However, in evaluating the detection of sliding signs for intra-abdominal adhesions, transabdominal ultrasound presents several advantages over transvaginal ultrasound, particularly in terms of patient comfort and broader applicability. A transabdominal ultrasound (TA US) is less invasive and generally more acceptable to patients, especially those who may have cultural or personal reservations about internal examinations. This non-invasive approach not only enhances patient compliance but also reduces the risk of infection associated with internal probes. Moreover, TA US offers a broader field of view, allowing for comprehensive assessment of abdominal and pelvic adhesions, which is crucial in cases where adhesions are extensive or the anatomical spread is uncertain. This method is particularly beneficial in diverse clinical settings, including those where transvaginal access might be contraindicated or technically challenging, such as in patients with severe vaginal atrophy or in early stages of pregnancy. The wider applicability and ease of use of TA US, coupled with its comparable diagnostic accuracy, make it a valuable tool in the preoperative assessment of patients with a history of cesarean sections. These advantages underscore the utility of transabdominal ultrasound in enhancing surgical planning and improving patient outcomes in obstetric and gynecologic practices.

In our study, intraoperative adhesions were confirmed in 16 of 35 patients (45.7%), aligning with previous reports that place adhesion incidence after repeat CS between 30–60% depending on parity and surgical history. Interestingly, although there were no significant differences in age or obstetric status between adhesion-positive and -negative groups, a significant association was found with the number of prior CS (*p* = 0.030). This supports existing evidence that prior uterine surgeries are a key driver of adhesion formation due to repeated peritoneal disruption, inflammation, and collagen deposition. In analyzing the diagnostic utility of the sliding sign, we also found that neither age nor gravidity significantly affected the likelihood of positive or negative sliding signs in both TA and TV groups. However, in the transvaginal group, there was a statistically significant association between the number of prior CS and the sliding sign result (*p* = 0.014), supporting the hypothesis that adhesions detectable *via* this technique may localize in the lower anterior pelvis, particularly in patients with repeated uterine access.

Regarding incision type, we observed a higher proportion of adhesions in patients with Pfannenstiel incisions (81.3%) compared to those with midline (mediana) incisions (18.8%). However, this difference was not statistically significant (*p* = 0.312), and the wide confidence interval (OR = 4.154; 95% CI [0.387–44.566]) suggests that this result may be due to underpowering, especially given the small number of midline cases. Our findings align with previous literature indicating that the type of incision may not be the primary determinant of adhesion formation, particularly in obstetric surgeries. In a review by Awonuga et al. (2011), it was noted that the presence of adhesions following cesarean section or gynecologic laparotomy was more strongly influenced by the surgical indication and tissue trauma rather than the incision type itself. Supporting this, Brill et al. (1995) found that patients who underwent gynecologic surgery—regardless of whether a midline or Pfannenstiel incision was used—had higher rates of adhesions than those who underwent obstetric surgeries, suggesting that surgical complexity and procedure type play a larger role than incision orientation per se. Furthermore, other studies emphasize that adhesiogenesis is multifactorial, involving peritoneal trauma, ischemia, foreign body reaction (*e.g.*, to sutures or talc), and surgical technique. One critical technical factor is peritoneal closure. Evidence from a Cochrane review by Bamigboye & Hofmeyr (2014) concluded that non-closure of the parietal peritoneum during cesarean section was associated with a higher incidence of adhesions at subsequent surgeries, suggesting that surgical strategy and technique may be more modifiable contributors to adhesion development than incision type alone.

One of the most striking findings in this study was the strong association between keloid formation and intra-abdominal adhesions. Among patients with visible keloids, 70% had adhesions, compared to only 13.3% in the non-keloid group (*p* < 0.001, OR = 15.167). This supports the hypothesis that keloid-prone individuals may have a systemic predisposition to exaggerated fibrotic responses, likely driven by increased fibroblast proliferation, dysregulated transforming growth factor-beta (TGF-*β*) signaling, and prolonged wound healing. This connection between external scar phenotype (*e.g.*, hypertrophic or keloid scar) and intra-abdominal adhesiogenesis has been previously proposed in both dermatologic and gynecologic literature. A prospective study by Tulandi et al. (2011) directly supports this association, reporting that women with keloids were significantly more likely to have dense adhesions—particularly between the uterus and the bladder (*p* = 0.028) and between the uterus and the anterior abdominal wall (*p* < 0.0001). These findings provide biological plausibility and external validation for our observation that visible cutaneous keloids may reflect an underlying pro-fibrotic phenotype affecting both cutaneous and peritoneal tissues. Thus, our study adds to a growing body of evidence suggesting that visible keloids may serve as an additional practical clinical marker for predicting intraoperative adhesion risk, potentially informing preoperative counseling, surgical planning, and operative preparedness in women undergoing repeat cesarean deliveries.

Intraabdominal adhesions have far-reaching consequences that extend beyond the technical challenges they pose during repeat cesarean delivery. Adhesions are a major contributor to chronic pelvic pain, bowel obstruction, infertility, and ectopic pregnancy, and they are associated with increased risks in future pregnancies, including abnormal placentation (*e.g.*, placenta accreta spectrum), uterine rupture, and peripartum hemorrhage. These complications can adversely affect patient quality of life, increase healthcare costs, and require more complex surgical interventions. Given the significant maternal morbidity linked to adhesions, preoperative identification using non-invasive and accessible methods such as the sliding sign becomes crucial. Our findings suggest that both transvaginal and transabdominal sliding sign evaluations may not only inform operative planning but also serve as predictive tools for long-term reproductive outcomes, facilitating early counseling and risk stratification. This underscores the broader clinical utility of incorporating sliding sign assessments into routine third-trimester ultrasound evaluations for women with prior cesarean deliveries.

This study has several strengths. The within-subject design, in which all participants underwent both transabdominal and transvaginal sliding sign assessments, allowed for a robust direct comparison between modalities while minimizing confounding factors. Additionally, the blinded ultrasound assessment and the use of intraoperative findings as the reference standard ensured a high level of diagnostic reliability. A unique strength of this study is the novel exploration of the relationship between keloid formation and intra-abdominal adhesions, offering early evidence that visible external scarring may serve as a clinical marker of adhesion risk. However, several limitations should be acknowledged. The sample size was relatively small, potentially limiting the statistical power, particularly in subgroup analyses such as incision type. The absence of body mass index (BMI) data may confound both sonographic interpretation and adhesion formation. Adhesions were classified as present or absent without the use of a standardized scoring system, which precluded analysis of severity. Furthermore, the study was conducted at a single tertiary referral center, which may affect generalizability. Finally, the absence of operative outcome data—such as dissection time, estimated blood loss, or intraoperative complications—limits the ability to fully contextualize the clinical impact of the ultrasound findings. Future multicenter studies with larger cohorts and standardized adhesion scoring are recommended to validate and expand upon these findings.

## Conclusions

Transabdominal and transvaginal sliding sign have good accuracy in detecting intra-abdominal adhesions in third-trimester pregnant women with a history of caesarean section.

##  Supplemental Information

10.7717/peerj.20551/supp-1 Supplemental Information 1 Raw data

10.7717/peerj.20551/supp-2 Supplemental Information 2 STROBE checklist

## Additional Information and Declarations

### Data Availability

The following information was supplied regarding data availability: The raw data are available in the Supplemental Files.
